# Hartmann’s Boundaries Questionnaire: Measuring Psychometric Properties and the Structure in a Russian-speaking Sample

**DOI:** 10.11621/pir.2022.0207

**Published:** 2022-06-30

**Authors:** Anna S. Kamardina

**Affiliations:** a Lomonosov Moscow State University, Moscow, Russia

**Keywords:** Psychological boundaries, boundaries questionnaire, validation, reliability, factor analysis

## Abstract

**Background:**

Psychological boundaries are defined as one’s ability to distinguish various components of psychological life: conscious and subconscious, self and others. The Boundaries in the Mind concept by E. Hartmann belongs to the psychodynamic tradition and is implemented in the Boundaries Questionnaire, which assesses the thinness of one’s psychological boundaries. Its shortest version (18 items) has been adapted in Russian but is a one-scale tool. The BQ-46 version, developed by D. Rawlings, consists of six subscales which allow for structural analysis.

**Objective:**

The objective of this study was to develop a Russian version of the questionnaire based on the BQ-46 and analyze its structure in a Russian-speaking sample.

**Design:**

The BQ-46 was translated into Russian and back. Data collection was arranged online. Three hundred eighty-seven (387) participants filled in the Russian version of the questionnaire. Two hundred ninety-six (296) of them also filled out four additional questionnaires for convergent validity assessment, and one hundred and one (101) subjects completed a BQ-46 retest.

**Results:**

The Russian version of the questionnaire (referred to as BQ-33) consists of 33 items and is a five-scale measurement tool. Its subscales are generally in line with the original BQ-46 version by D. Rawlings. The BQ-33 demonstrated acceptable-to-good reliability and good test-retest stability (r = 0.86). The convergent validity of the BQ-33 was supported by associations with the respective psychological concepts. My findings supported the idea of boundaries getting thicker with age, along with some tendency for gender differences for particular subscales.

**Conclusion:**

The study results supported the validity and reliability of the BQ-33 in a Russian-speaking sample. This tool can be used to measure psychological boundaries and assess their structure.

## Introduction

Psychological boundaries are usually defined as the ability to distinguish between 1) self and others (external boundaries) and 2) various components of psychological activity (internal boundaries). Strengthening one’s boundaries is often one of the most important goals of a psychotherapy process. Nevertheless, there is a tendency to judge boundaries by qualitative methods only; this is supported by the history of the concept’s development.

The concept of psychological boundaries has been being elaborated since the early 20th century. Each of the psychological approaches made its own contribution to the process. Tausk (1919) was the first to apply the term as a metaphor to describe patients with schizophrenia and their losing a sense of self. P. Federn in his work “Narcissism in the Structure of the Ego” advanced understanding of the concept by discriminating between internal and external boundaries, and stating that the boundaries are not just a personal trait but also a characteristic of one’s state during a day (Gabbard & Lester, 2014).

The substantial contribution of the psychodynamic approach lies in the area of boundaries’ development: for example, the process of separation-individuation described by M. Maler shed light on how a baby overcomes psychosomatic unity and gets his or her own boundaries, physical and psychological (Maler et al., 2011). Boundaries dysfunction was implied in Kernberg’s determination of identity diffusion and reality testing, two of the three criteria for assessing the level of personality organization (Kernberg, 2019). Hartmann’s concept of Boundaries in the Mind, having evolved from studying the individual differences of people with nightmares (Hartmann & Kunzendorf, 2006), added perceptual sharpness to the classical psychodynamic understanding (Gabbard & Lester, 2014; Harrison & Singer, 2013).

In his Field theory, K. Levin formulated the idea of the boundary of contact (Levin, 2000), which was further developed by F. Perls in gestalt theory (Perls, 2020, 2005). This theory defines a boundary as a place of contact, which performs two opposite but united functions: differentiating and interrelating. Understanding the ambivalent nature of boundaries is crucial. As Tkhostov (2002, p.78) asserted: “Self is located exactly where not-Self starts.” Perls and his followers have also described several mechanisms of boundaries’ dysfunction (Ginger & Ginger, 2017).

Since psychological boundaries were initially understood metaphorically, the first attempts to study them were mostly qualitative. Interviews and projective techniques provided an understanding of how boundaries dysfunction could be manifested. The next step was the attempt to move from an idiographic to a nomothetic approach by estimating the qualitative data quantitatively. For example, Fisher and Cleveland designed a test for estimating body image boundaries based on Rorschach protocols (Schizophrenia Research Trends, 2008; Sokolova, 2015). But still, there was a need for a more objective method of estimation, and the questionnaires were developed to address this demand.

To the best of my knowledge, the following questionnaires for psychological boundaries measurement have been validated for Russian speakers: 1) the I-structure test by G. Ammon (ISTA) (Kabanov & Neznanov, 2003), which estimates inter alia external and internal self-delimitation via three dimensions: constructive, deficit, and destructive components, or states; 2) the Boundaries questionnaire by N. Brown (Shamshikova, 2009), which assesses one’s behavior towards others’ boundaries; and 3) a short 18-item version of the Boundaries questionnaire by E. Hartmann (Shamshikova & Volokhova, 2013) using his Boundaries in the Mind concept.

In addition, Nartova-Bochaver has developed the Personal Sovereignty Questionnaire-2010 (Nartova-Bochaver, 2017). The concept of sovereignty corresponds to one of the psychological boundaries, although it is not exactly equal to it: psychological sovereignty is the ability to keep one’s boundaries and is expressed in six dimensions that are developed sequentially during a lifetime: body, territory, things (belongings), routine habits, social contacts, and tastes and values. T.S. Levi considers boundaries a functional agency and assesses them via her own questionnaire, which is structured by six subfunctions of the boundaries (Levi, 2017).

In this work, the focus is on Hartmann’s Boundaries in the Mind concept and aims to validate a shorter version of the questionnaire on the basis of the 46-item version (BQ-46) developed by D. Rawlings (2001–2002), which is, in its turn, based on Hartmann’s original 138-item-12-scale questionnaire. The BQ-46 is more time-consuming than the short 18-item version (Schredl et al., 2009) already validated in Russian (Shamshikova & Volokhova, 2013), but it provides the benefit of allowing structural analysis because it has six subscales, while the short 18-item version is a one-scale technique.

The aim of this study was to solve the following tasks: 1) to develop a Russian version of the questionnaire based on the BQ-46; 2) to assess its psychometric characteristics in a Russian-speaking sample; and 3) to perform factor analysis in order to define the construct’s structure and the associations of its components with other psychological concepts.

## Methods

### Participants

The subjects were recruited via social networks and among MSU students in Masters and PhD programs. There were 387 participants (net of subjects excluded based on the “lies scale” results as described below); the female/male ratio was 73% to 27%, and the subjects’ ages ranged from 16 to 67 (M = 36.1, SD = 9.2). Most of participants had higher education (87%, plus 3% with a PhD or Doctorate degree).

Fifty-six subjects (14%) mentioned that they had sought out a psychiatrist with regard to their mental condition, and 13 of them said they had been diagnosed.

### Procedure

The questionnaire underwent several iterations of English-to-Russian and back translations with professional translators involved in order to ensure the wording captured the respective phenomena. The final version was agreed upon with D. Rawlings.

Data collection was arranged via a tailor-made website. As soon as the participants confirmed their age and provided their informed consent for participation in the research, five questionnaires were available for sequential completion. To motivate the participants, I provided them with automatic feedback based on the Big Five Personality test. To those who left their emails, the questionnaire was available for retesting five weeks after the first completion date. One hundred and one subjects agreed to participate in the retest.

The data was collected in three stages:

1. The preliminary stage: March-April, 2021. N = 36. Subjects’ feedback on the questionnaire wording and website usability was collected, which resulted in rephrasing several items and addressing a few technical issues;
2. The main stage: May-October, 2021. N = 260. Data collection for correlation analysis and test-retest stability assessment.
3. The final stage: November 2021 — February 2022. The sample was enlarged for the questionnaire structure analysis, with only BQ-46 being presented. N increased to 387.

After the participants in the first two stages filled out the questionnaires containing close-in-meaning items, their answers were submitted to a “lies scale” test; those who provided opposite answers to nearly identical questions more than once were eliminated from the database. For the first two stages, 11 pairs of items were used for the comparison, with the result that seven subjects were excluded.

### Questionnaires

The original version of the questionnaire (Rawlings, 2001–2002) consists of 46 statements with a 5-point Likert-type scale ranging from 0 (“Not agree at all”) to 4 (“Totally agree”). Twenty-two out of 46 items are reverse scored. Items are allocated to six subscales, which are presented in *Table 1.* The total score is a sum of the first five subscales, while the Trust subscale is not included, as it reported a negative correlation with the total. The highest scores indicate thinner boundaries.

**Table 1:** Subscales and item examples of the original BQ-46 questionnaire

Subscale	Item Examples
Unusual Experience (UE)	24. My body sometimes seems to change its size and shape. 39. I have often had the experience of different senses coming together. For example, I have felt that I could smell a color, or see a sound, or hear an odor.
Need for Order (NFO)	2. *In an organization, everyone should have a definite place and a specific role.* 13. *I like stories that have a definite beginning, middle, and end.*
Childlikeness (Ch)	11. Children and adults have a lot in common. They should give themselves a chance to be together without any strict roles. 19. I think a good teacher must remain in part a child.
Perceived Com- petence (PC)	6. *I keep my desk and worktable neat and well organized.* 10. *I get to appointments right on time.*
Sensibility (Se)	9. I am easily hurt. 18. I am a very sensitive person.
Trust (Tr)	31. I am a very open person. 37. Sometimes I meet someone and trust him or her so completely that I can share just about everything about myself at the first meeting.

*Note. In italic are reverse scored items*

The following questionnaires validated in Russian were used to estimate the convergent validity of the questionnaire:

1. The short 18-item version of the Boundaries questionnaire by E. Hartmann (Shamshikova & Volokhova, 2013);
2. The scales of Internal and External self-delimitation of the I-structural test by G. Ammon (ISTA) (Kabanov & Neznanov, 2003);
3. The boundaries questionnaire by N. Brown (Shamshikova, 2009); and
4. The Big Five Personality Test (Khromov, 2000).

### Analysis

The data was processed in IBM SPSS Statistics 27, Jamovi 1.6.21, and FACTOR (Lorenzo-Seva & Ferrando, 2006).

For correlation analysis I used Spearman’s ratio (for subscale correlation and to confirm the questionnaire’s convergent validity) and polychoric correlation (more suitable for ordinal variables (Holgado-Tello et al., 2010) and, thus, used for item correlation matrix for factor analysis). In analyzing the structure of the questionnaire, I performed exploratory factor analysis (EFA) to elaborate the initial model and unrestricted confirmatory factor analysis (CFA) incorporated in FACTOR (RETAM) by Lorenzo-Seva & Ferrando (2020), both based on polychoric correlation matrices, if not stated otherwise. An ANCOVA procedure was used to assess the effect of age and gender factors on the boundaries.

Conclusions were based on a commonly accepted level of significance at 0.05, if not stated otherwise.

As a few of the participants stated they had a psychiatric diagnosis, I analyzed the data both altogether and separately for those who didn’t mention any diagnosis. I also performed an ANOVA procedure for subjects with and without diagnoses, as well as for ones with and without the experience of seeing a psychiatrist. As no differences were found, I report the results of the sample without reference to the participants’ diagnoses and seeking psychiatric help.

## Results

### Questionnaire Content, Structure, and Reliability

No duplicate items were found since there was only one pair of items which showed a correlation level over 0.6 (items #19 and #23 correlated at 0.715), while only 13 pairs demonstrated a correlation level over 0.50.

Based on the complexity analysis, item #7 (“A good teacher ought to help the child to remain special”) was excluded since 97% of the responses were positive (“Totally agree” or “Rather agree”). Additionally, two items (#1 “I am careful about what I say to people until I get to know them really well” and #5 “I think I would make a good psychologist”) were candidates for removal based on the breach of above 0.5 Measure of Sampling Adequacy (MSA) criterion (normed MSA reported at 0.40 and 0.39 respectively, N = 387) (Lorenzo-Seva & Ferrando, 2021).

Factor analysis suitability was confirmed by the correlation matrix of the questionnaire items (almost no strong correlations as mentioned above) along with the Kaiser-Meyer-Olkin (KMO) MSA (0.746 > 0.5) and Bartlett’s Test of Sphericity (χ^2^ = 4269, df = 903, p <0.001) calculated for the 43-item version.

As the sample size was limited, given the number of questionnaire items, presented are the results of factor analysis for N=387. Two subsamples randomly split off from the dataset had KMO MSA at 0.535 and 0.586 respectively, close to the acceptable threshold of 0.5, which supports the idea of declining sample split in this study. Meanwhile, results of EFA performed on one subsample, and a RETAM procedure performed on the other, are in line with the results presented below (please refer to the Note at the end of this article for details).

In the first stage, a model based on EFA (Robust Unweighted Least Squares in combination with promin rotation, based on polychoric correlation matrix) performed for the 43-item version of the questionnaire was constructed. Parallel analysis supported a five-factor model, explaining 42.0% of data variance, while the scree plot criterion provided six factors (45.6% of data variance), which was exactly in line with the original questionnaire’s structure by D. Rawlings (2001–2002). Thus, further analysis started with these two models and, based on the items’ allocation, the original names of the factors were kept; *i.e.,* Unusual experience (UE), Need for order (NFO), Childlikeness (Ch), Perceived competence (PC), Sensibility (Se), and Trust (Tr). The models had almost identical structures, with the only difference being that the NFO and PC factors of the six-scale model were combined into a single factor in the five-scale version. Further elaboration of the models by removing the items that could not be allocated definitively to a single factor, or with factor loadings below 0.3 (almost the same list of items for the both models except for #6 removed from the six-factor model and kept in the five-factor one), left us with just the five-factor model, as the PC factor items of the six-factor model needed to be either removed or reallocated to the NFO factor.

*Table 2* presents further development of the five-factor model conducted with FACTOR. Further removal of “problem” items (with low or ambiguous factor loadings) naturally increased the Model fit ratios. As FACTOR incorporates the option of unrestricted factor analysis using RETAM as a procedure for objectively refining target matrices (Lorenzo-Seva & Ferrando, 2020), the number of changes to the loading matrix proposed by RETAM was used as a criterion to choose the final version of the model. Thus, the analysis ended up with Model C, further referred to as the BQ-33, representing the final version of the questionnaire derived from the Russian-speaking sample.

**Table 2:** Five-factor models’ statistics performed by FACTOR (EFA and RETAM results)

Model	N of factors (n of items)	Sample Statistics			Model Fit Ratios (EFA)					RETAM results
		KMO test	Bartlett’s statistic	df	χ^2^**	df	CFI	BIC	RMSEA	
A	5 (38)	0.752	4 289*	703	721.8*	523	0.978	2 080	0.031	four changes introduced
B	5 (34)	0.767	4 304*	561	531.1*	401	0.983	1 747	0.029	three changes introduced
C	5 (33)	0.768	4 308*	528	495.6*	373	0.983	1 675	0.029	no changes introduced

Note. * p < .001, ** Robust Mean and Variance-Adjusted Chi Square

**Figure 1.**
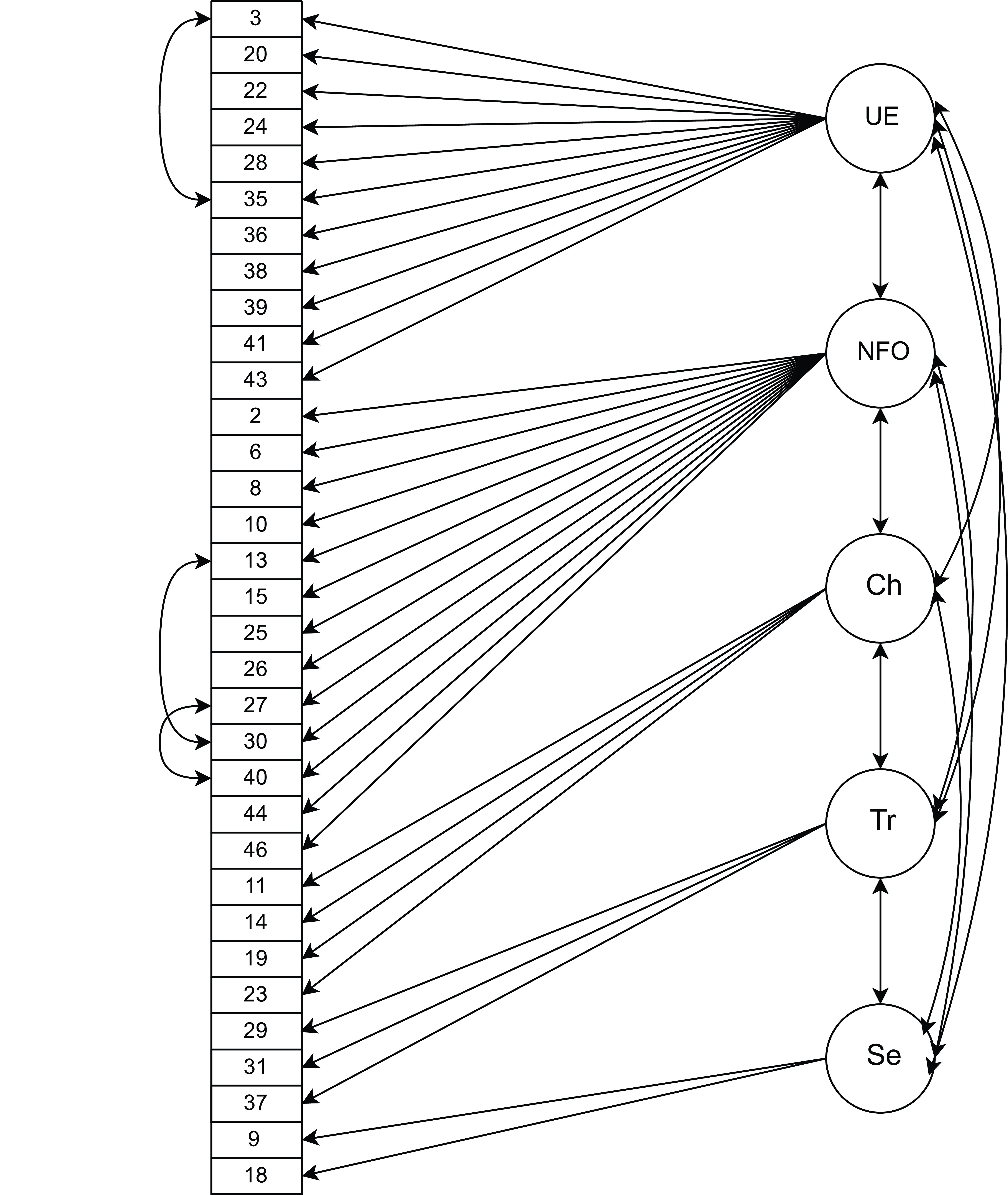
Model C’ structure (five factors, 33 items, three pairs of residual covariances)

I further elaborated the model using the CFA procedure developed by Jamovi (based on Pearson’s correlation matrix since this is the only option incorporated in the program) to assess the effect of adding residual covariances of three pairs of items. I included only those pairs for which such covariances can be theoretically supported by the items’ content (items of the first pair were about nightmare experience; items of the second pair were about being neat; items of the third pair had the same phrase structure: #27 “East is East, and West is West…” and #40 “A man is a man, and a woman is a woman…”). This step led to an increase in the model’s CFI from 0.847 to 0.873 and a decrease in RMSEA from 0.0465 to 0.0425. The results are reported for models’ comparison purposes only since the main factor analysis was performed based on a polychoric correlation matrix in accordance with the data type, as described above. *Figure 1* presents the final model structure.

In the next stage, Model C factors were analyzed. *Table 3* summarizes the results of correlation analysis along with the factors’ reliability ratios. According to Ferrando & Lorenzo-Seva (2018), the 0.9 threshold for Factor Determinacy Index should apply in the factor score assessment (while the 0.8 one is still suitable for research purposes). A congruence value over 0.85 corresponds to a fair similarity (between a rotated loading matrix and a target matrix), while a score over 0.95 implies that the compared factors can be considered equal (Lorenzo-Seva & ten Berge, 2006). These criteria supported good reliability of Model C factors. The weaker ratios of the Se factor may have come from the fact that it was composed of just two items (although in line with the original model by D. Rawlings).

**Table 3:** Factor reliability and correlation matrix based on 5-factor/33-item Model C

Factor	Factor Determinacy Index	Congruence Ratio*	Inter-Factors Correlation Matrix				
			UE	NFO	Ch	Se Tr
UE	0.943	0.975	1.000			
NFO	0.921	0.965	0.001	1.000		
Ch	0.931	0.931	**0.217**	–0.064	1.000	
Se	0.862	0.886	0.168	–**0.225**	0.109	1.000
Tr	0.877	0.913	0.122	–0.119	0.148	**0.235** 1.000

Note. * Congruence between rotated loading matrix and target matrix; in bold are correlation ratios with an absolute value over 0.20

While a low KMO test score (0.567) does not fully support second level factor analysis, based on the correlation matrix, the following allocation of the subscales to three broader dimensions is possible to assume:

- Subscales UE and Ch relate to the Intermixture dimension;
- The NFO subscale represents the Order dimension;
- Subscales Se and Tr relate to the Relations dimension.

This hypothesis was verified with a CFA procedure performed with Jamovi (based on a Pearson correlation matrix supported by below 1.0 skewness and kurtosis ratios for all of the five subscales except for UE kurtosis at 1.2). The model fit ratios did not support the hypothesis: χ^2^ = 4.33, df = 3, p = 0.228, while CFI = 0.975 was high enough, the RMSEA 90% CI was between 0.000 and 0.098. Thus, the second level structure needs further investigation.

### Test-Retest Stability

The test-retest stability of the questionnaire and its subscales was assessed by a Spearman’s rank correlation ratio for the average retest period of 48.5 days (SD = 9.5). *Table 4* summarizes the results, which supported the stability of the BQ-33 and its subscales in short-term run.

**Table 4:** Test-Retest Reliability for BQ-33 (5-factor/33-item version)

Subscale	Test-retest correlation ratio
UE	0.804*
NFO	0.833*
Ch	0.682*
Se	0.824*
Tr	0.752*
BQ-33	0.864*

Note. * p < .001

### Convergent Validity

Correlation analysis was applied to assess the questionnaire’s convergent validity. Here I used Spearman’s rank correlation ratios since the skewness and kurtosis tests allowed this. *Table 5* shows the respective correlation ratios reported based on the data of 296 participants.

**Table 5:** BQ-33 and subscale correlation ratios with other psychological concepts (within convergent validity assessment), N=296

	BQ–33	Subscales				
		UE	NFO	Ch	Se	Tr
BQ–33	–	0.744 ***	0.520 **	0.342 ***	0.122 *	0.352 ***
Boundaries (Hartmann’s Q18)	0.662 ***	0.569 ***	0.175 **	0.347 ***	0.348 ***	0.244 ***
Boundaries (N. Brown Q)	0.284 ***	0.222 ***	0.085	0.163 **	0.030	0.137 *
I–structural test by G. Ammon						
External Constructive	–0.203 ***	–0.189 **	–0.067	0.044	–0.321 ***	0.020
External Destructive	0.092	0.186 **	–0.091	0.052	0.174 **	–0.121 *
External Deficit	0.352 ***	0.294 ***	–0.070	0.141 *	0.573 ***	0.305 ***
Internal Constructive	–0.238 ***	–0.108	–0.203 ***	0.010	–0.270 ***	0.005
Internal Destructive	–0.069	0.027	–0.168 **	–0.026	0.193 ***	–0.084
Internal Deficit	0.479 ***	0.443 ***	0.056	0.157 **	0.431 ***	0.218 ***
Big Five Personality Test						
Extraversion	0.265 ***	0.198 ***	0.063	0.174 **	0.005	0.282 ***
Agreeableness	0.015	–0.050	–0.074	0.106	0.039	0.253 ***
Control (conscientiousness)	–0.486 ***	–0.238 ***	–0.448 ***	–0.120 *	0.012	–0.132 *
Emotionality (neuroticism)	0.262 ***	0.263 ***	–0.121 *	0.077	0.675 ***	0.163 **
Playfulness experience) (openness to	0.326 ***	0.272 ***	0.085	0.215 ***	0.123 *	0.166 **

Note. * p < .05, ** p < .01, *** p < .001

The total scores on the BQ-33 showed a high correlation with the boundaries scores assessed by the short (18 items) version of Hartmann’s questionnaire: a Spearman’s ratio of 0.66, p < 0.001. While the ratio was supposed to be supported by seven very-close-in-meaning items in the two questionnaires, four of these items belonged to the NFO subscale, which reported the lowest correlation ratio with the short version total scores.

A much lower, but still significant correlation was reported between the BQ-33 total scores and the boundaries scores measured by the N. Brown questionnaire. Interestingly, when assessed by particular subscales, the total scores on the N. Brown questionnaire reported being associated with the subscales of the Intermixture dimension (EU and Ch), rather than with the Relations ones (Se and Tr) as might have been expected based on the fact that the N. Brown questionnaire measures boundaries in interpersonal interaction.

The BQ-33 reported a lack of associations with the destructive scales of the I-structural test, both internal and external self-delimitation, while deficit functions were the most strongly correlated with the BQ-33 total score. The general trend was that the thinner the boundaries, the more deficit and less constructive was self-delimitation.

Among the Big Five traits, the one most associated with the boundaries’ character was Control (or the more generally accepted term, Conscientiousness): subjects with thinner boundaries reported less control. As expected, the NFO was among those most strongly associated with Conscientiousness. Neuroticism, Openness to experience, and Extraversion were positively correlated with the BQ-33 results, while Neuroticism was strongly associated with the Se subscale.

### Descriptive Statistics and Age & Gender Analysis

**Figure 2.**
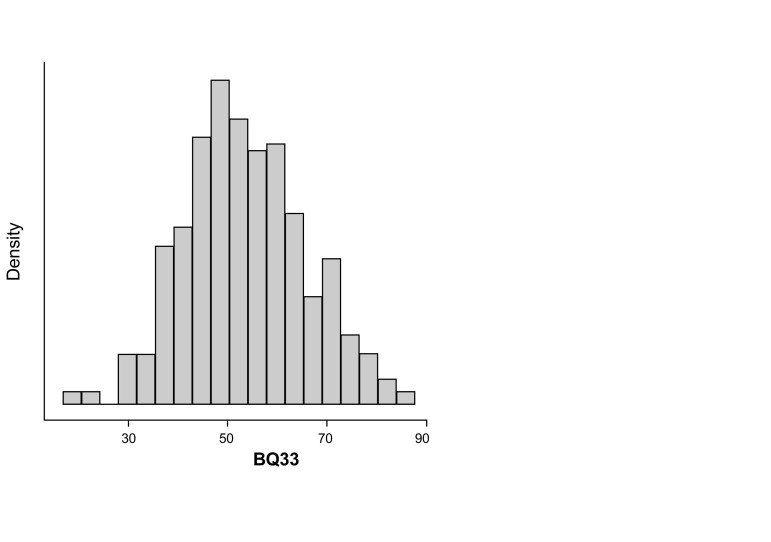
Distribution of the BQ-33 total score (N=387)

The mean of the BQ-33 scores was 53.3, SD = 12.1. It was normally distributed, which is shown by *Figure 2* and confirmed by the Shapiro-Wilk test (W = 0.994, p = 0.133), and the skewness and kurtosis test (both did not exceed the respective standard error: skewness = 0.124, SE_skewness_ = 0.124; kurtosis = –0.106, SE_kurtosis_ = 0.247).

**Figure 3.**
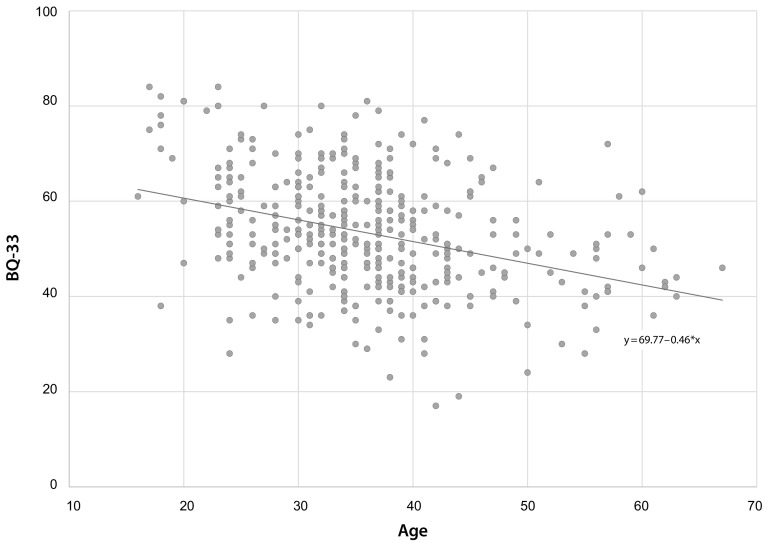
Scatter plot of BQ-33 total scores over subjects’ age (N=387)

An ANCOVA procedure was applied to assess the boundaries’ associations with age and gender. The interaction of the factors had no statistically significant impact so only main effects were analyzed. Statistically significant differences were reported for age only: F = 52.18, p<0.001, partial ŋ^2^ = 0.12. The findings corresponded with the idea of boundaries getting thicker with age (please refer to the scatter plot in *Figure 3*), while the hypothesis about females tending to have thinner boundaries was not supported (the mean for females was 53.6, which exceeded the mean for males at 52.6, but no statistically significant differences were reported).

The same ANCOVA procedure applied to various boundaries subscales revealed that only the UE (F = 33.24, p < 0.001, ŋ^2^p = 0.080) and NFO (F = 33.58, p < 0.001, ŋ^2^p = 0.080) subscales were associated with age, while gender contributed to statistically significant differences in the Se (F = 32.17, p < 0.001, ŋ^2^p = 0.077) and Ch (F = 9.40, p = 0.002, ŋ^2^p = 0.024) subscales. All the tests were performed for main effects only, but the effect sizes are low. While age contributed to the boundaries’ thickness along both the subscales and the total, gender affected the subscales multi-directionally: females reported higher scores (thinner boundaries) in the Se subscale and lower scores in the Ch subscale, which resulted in no effect of the gender factor on the total score.

## Discussion

There are several questionnaires to estimate psychological boundaries, both adapted for a Russian-speaking population and initially developed by Russian psychologists. Most of them are structured according to boundaries’ functions (Kabanov & Neznanov, 2003; Levi, 2017) or represent a one-scale technique (Shamshikova, 2009; Shamshikova & Volokhova, 2013) thus precluding the possibility for structural analysis. The aim of this work was to adapt the short version of the boundaries questionnaire developed by D. Rawlings (2001–2002) within the Boundaries in the Mind approach by E. Hartmann and to analyze its structure in a Russian-speaking sample.

A five-scale model of the boundaries concept was developed. It is in line with the structure of the original questionnaire by D. Rawlings, while merging two of the subscales of the original questionnaire into a single factor. The subscale names were kept the same based on the content of items: Unusual experience (UE); Need for order (NFO, merged with Perceived Competence items from the original questionnaire); Childlikeness (Ch); Sensibility (Se); and Trust (Tr). While D. Rawlings reported a negative correlation of the Trust subscale with the total score on the questionnaire and thus excluded that subscale from the final boundaries score, in the Russian-speaking sample a statistically significant positive correlation of the ratios was reported, which resulted in keeping the Trust subscale in the final structure.

The second-level analysis of the subscales led to the idea that they can be further arranged into three dimensions — *i.e.,* Order (NFO), Intermixture (UE and Ch), and Relations (Se and Tr) — but this still requires further verification since no supportive factor analysis results were reported for the sample of this study. The two-level structure has been reported by D. Rawlings as well and may form a basis for further development of the boundaries concept from a hierarchical point of view.

The total scores on the BQ-33 were strongly positively correlated with the scores on the existing Russian version of the short 18-item Hartmann’s boundaries questionnaire, since both questionnaires come from the same boundaries concept. An association with the boundaries score on the N. Brown questionnaire was reported, but the correlation ratio was weaker, which can be explained by the fact that Brown’s questionnaire measures one’s ability to keep a distance in interpersonal interaction and avoid violation of the other’s private space. This is just one aspect of a relationship which is the product of numerous factors to which psychological boundaries contribute only a part.

I also compared two constructs: Hartmann’s psychological boundaries and self-delimitation as determined by G. Ammon (Ammon, 2019; Kabanov & Neznanov, 2003). Both were within the psychodynamic approach to psychological boundaries. Ammon considers delimitation in two dimensions: 1) internal and external, and 2) constructive, destructive, and deficit. The second set of criteria identifies three states that are intercorrelated but still represent a particular aspect of boundaries functioning: a) a constructive state is associated with one’s ability to distinguish self from others, to separate some aspects of inner psychological life from others (dreaming, fantasies and reality, past and present), to keep the balance of involvement into and distance from the world, and to successfully integrate one’s experiences; b) a destructive state corresponds to rigid closure of one’s and others’ emotions and needs, a lack of fantasy and dreaming as a result of poor access to the subconscious, and putting a priority on the rational over the emotional; and c) a deficit state is associated with the lack of distinction between conscious and subconscious areas, the lack of ability to distinguish one’s self from others, and the predominance of, or even capture by, feelings.

My initial understanding of the interaction of these concepts was that the Rawlings’ questionnaire should provide boundaries scores from the most destructive state (the thickest boundaries which do not allow interaction between the rational and emotional, or conscious and subconscious areas) to the most deficit one (the thinnest boundaries without any distinction at all). But the findings did not fully support that initial view, since only low correlations of the total boundaries scores with destructive states for both internal and external self-delimitation were reported. The results of the analysis supported the view that BQ-33 measures psychological boundaries from a more constructive end (lower scores) to a more deficit one (higher scores), but ignores the destructive state to some extent.

When compared with the Big Five traits, the higher boundaries’ scores (thinner boundaries) were associated with lower consciousness, which can be viewed as evidence that the boundaries concept is related to volitional regulation of behavior. On the other side, higher scores on the boundaries questionnaire correspond to higher neuroticism, openness to experience, and extraversion.

This study findings supported the idea of psychological boundaries becoming thicker with age. This corresponds to theoretical views of boundaries development throughout a person’s entire life. Although a person’s first years are still extremely crucial for the process (Maler et al., 2011; Storck, 2005), boundaries keep developing in response to the environment in adolescence and afterward (Nartova-Bochaver, 2005; Nartova-Bochaver & Silina, 2009). The results of this study are well in line with international researchers’ findings.

One should be cautious about considering the results of the gender analysis. While some subscale scores were varied by gender, the effect sizes were rather low for drawing any conclusions.

## Conclusion

The Russian version of the Boundaries Questionnaire (BQ-33) has been developed, consisting of 33 items arranged into five subscales with acceptable-to-good reliability and good test-retest stability. Convergent validity was confirmed by comparing the total and scale scores with the respective psychological concepts. This tool can be used to measure psychological boundaries and assess their structure.

Further research should aim at increasing the sample in order to reconfirm the questionnaire’s structure on a broader population and develop standards for different age groups. Future studies might also involve the clinical population and focus on the phenomenon’s specific characteristics for patients with various diagnoses.

## Limitations

This study was not without limitations:

1. Due to a limited number of subjects, I had to present the results of both EFA and CFA (semi-confirmatory factor analysis provided by FACTOR) performed on the same data, although it would have been more proper to split the sample into two parts, one for each type of analysis, to avoid overfitting, that is, to avoid receiving inflated estimates of model fit and parameter estimates (Fokkema & Greiff, 2017). The random split provided two subsamples with statistics not supportive for further factor analysis, though the results are presented in the Note at the end of this article and are in line with the findings presented above. Further replication of the structure should be tested based on a new data sample.

2. Since the participants were recruited via social networks and from Moscow State University students, the results should be extrapolated to the general population with some prudence. For example, Hartmann et al. (2001) have reported several studies supporting the idea of occupation preferences varying for people with different types of boundaries, so some occupation bias could be included in the results.

3. While assessing the influence of the age factor on BQ-33 scores, I should note that a longitudinal study would be more suitable for this assessment. The reported association of the boundaries score with age is replicable in other language samples, but still can be a result of external factors (like the cohort effect), at least to some extent.

## Note

To assess the effect of no sample-split on the results of factor analysis, it was conducted for two randomly split subsample despite their low KMO MSA measures. Subsample A with N = 197 subjects had an MSA score of 0.535. Subsample B with N = 190 had an MSA score of 0.586. S = KMO_A_/KMO_B_ = 0.913, the ANOVA procedure showed no significant differences in the total score of 43 items in the subsamples (F = 1.24, df1 = 1, df2 = 385, p = 0.266). EFA performed for Subsample A supported a five-factor model. Unrestricted CFA applied to Subsample B introduced six changes to the loading matrix, which resulted in the five-factor/30-item model, with the majority of items removed being the same as the ones removed in factor analysis applied to the whole sample. The allocation of the items to the factors stayed the same as the one of Model C retrieved from the whole data sample, with only one item migrating from the EU to the Se subscale. EFA and unrestricted CFA were conducted in FACTOR based on polychoric correlation matrices.
